# Safety in Housing for Older Adults—A Qualitative Case Study

**DOI:** 10.1177/07334648241260212

**Published:** 2024-07-18

**Authors:** Ira Verma

**Affiliations:** 1 Department of Architecture, 174277 Aalto University, Espoo, Finland

**Keywords:** safety, feeling of safety, older adults, housing

## Abstract

Housing solutions for older adults aim at providing a safe environment to live in. The construction is heavily based on aspects of physical safety, often disregarding the social aspects of feeling safe. The older adults themselves are looking for a living environment that supports their individual capacities and social networks. The living environment can contribute to their physical, social, and emotional safety by providing accessible housing and spaces for community building. Interviews with older adults who had recently lived through a building fire highlighted the importance of the community in daily life, in emergency as well as in recovery from hazards. The results show that community building is related to access to common-use spaces and daily interaction with neighbors. The shared spaces in the immediate surroundings can enhance community cohesion and generate peer support. The common-use spaces and public facilities in urban environment have a significant role in emergencies and in the process of recovery from adversities.

**What this paper adds**

• The paper provides further knowledge on the safety in housing experienced by the older adults.
• The results of this qualitative study show the importance of the community and communal spaces in the resilience and recovery from hazards.

**Applications of the study findings**

• The paper proposes practical measures to improve evacuation safety in housing for older adults.
• The results of the study highlight the importance of common-use spaces for building up social relationships and resilience.
• The study brings out need to involve older residents in home modifications and support their daily coping in temporary housing.

## Introduction

Current demographic development trends increase the need for housing environments that promote the daily living of urban residents with age-related frailty. In Europe, vast majority of older adults live in private households alone, with their spouse or with other persons. Older women are living alone more often than men (European Union, 2019, p. 32–34). For their home, older adults are looking for carefree, safe, and secure everyday life (Hasu, 2018). In the future, hazards related to our living environments are expected to increase and affect the most vulnerable groups of people.

Pendall et al. (2012) point out that vulnerability can be resulting from one’s living situations and related to one’s personal characteristics. Their study found that vulnerable people are more often living in inadequate housing conditions than other resident groups. Older adults may choose to remain living in housing, which is not ideal for their physical needs, but it is supporting their identity and continuity in life (Severinsen et al., 2016). Lau et al. (2007) argue that the declining functioning capacities and cognitive decline may become a risk for “aging in place,” remaining living at home in a familiar neighborhood. Dahlin-Ivanoff et al. (2007) found that older adults were associating living at home, in a familiar neighborhood to the feeling of safety. Perceived social safety, feeling of acceptance, belonging, and inclusion have benefits for their health and well-being (Slavich, 2020). Similarly, interviews by Pohjavirta (2021) revealed that people with memory decline considered their home environment, own routines, and familiar neighbors enhancing their feeling of safety.

Social safety is the result of interaction with other people in one’s living environment. Community accessibility may promote older adults’ social networks within the neighborhood. Kemperman et al. (2019) observed that the possibility to get to know new people and maintaining existing social networks was related to the quality of the built environment, access to local services, and facilities. Van Kessel (2013) found that the social inclusion of older adults and the ability to maintain effective relationships are critical in recovering from adversity. Physical environment, as a platform for social interaction, influences the capacity of older adults to be resilient. Social safety and networks of family, friends, and community also play an important role in home fire safety and assistance in fires. Coty et al. (2015) argue that the importance of these social networks for safety has not been discussed enough.

According to Dahlberg and McKee (2014), the emotional safety of older adults is a balance between the need for security and the fear related to the loss of control over one’s own life. It is connected to one’s self-esteem, experienced well-being, and having someone to turn to for help. Emotional safety of older adults with memory decline has been associated with perceived self-determination and, on the other hand, willingness to receive support (Grobosch et al., 2020). Hazards like building fires affect the sense of security and control over the home environment. The home environment, personal belongings, and own furniture are part of one’s personality. Heersmink (2018) found that if these meaningful and evocative personal objects are lost, it has implications for our memory, emotion, and self. There is evidence that best outcomes of home adaptations are achieved when users are involved in the decision-making process (Powell et al., 2017).

The efforts to improve safety at home should be realized with respect to resident’s personal freedom and the right of self-governance (Verma et al., 2022, p. 85). Home adaptations may affect the aesthetics of home and cause resistance from the part of the resident. Heywood (2005) argue that when home adaptations focus only on the functionality, ignoring the meaning of home, and the need of feeling in control, the benefits of such adaptations may be lost. Paque et al.'s (2018) findings show that losing self-determination due to, for example, institutionalization may decrease the feeling of security. A person may feel disempowered because of safety measures diminishing one’s sense of identity and worth (Sherwood-Johnson et al., 2019).

Feeling optimistic, having self-esteem, and self-efficacy are resilience resources, which can help older adults to better cope with stressful life events (Hajek & König, 2019). On the other hand, social isolation and cognitive decline are risk factors in hazard situations. The study by Karemaker et al. (2021) showed that older adults did not consider themselves as vulnerable or susceptible to the risk of a fire in their homes. According to their study, older adults may have a low risk perception and feel overconfident in their abilities to act in case of a fire. On the other hand, they were aware that their physical disabilities might prevent them from escaping or, for example, using the stairs in fire. Safety at home has been recognized as an under-researched topic (Kivimäki et al., 2019; Pettersson et al., 2020). Home safety for older adults has been studied more from the point of view of building safety and home care. Experiences of older adults living independently have received less attention.

This article addresses this gap and presents the experiences of five older adults who lived through a building fire. The author interviewed residents who were temporarily forced to move from their homes due to the fire and renovation process. The objective was to provide further knowledge on how to enhance physical, social, and emotional safety of older adults in hazard events. This study is part of a larger framework on future urban resilience focusing on the role of the built environment in aging cities. The paper gives a descriptive analysis of older adults’ experiences of safety and its relation to the built environment. The research question was how can housing environment and the built environment best support older adults’ safety and feeling of safety. The topic is important for cities undergoing demographic change.

## Background

### Functionality and User Friendliness

The need for safety, accessibility, and user-friendliness in the built environment is widely recognized. Housing construction for older adults and other vulnerable resident groups has specific requirements and safety regulations. These regulations aim at providing a safe environment to live in, trying to eliminate any risks. Existing guidelines are often limited to practical and normative aspects of housing construction, ignoring subjective aspects of user experience (Schaff et al., 2022). Due to increased longevity and aging in place policy, an increasing number of people will live at home at a very old age. Therefore, more attention needs to be on mainstream housing and community design supporting older population. In 2022 in Finland, 93% of older adults in the age cohort 75 years old and older were living at home. A majority of them (56 5 %) were living in one-person households (Sotkanet.fi, 2023).

Previous studies have shown evidence that old age, living alone, and functional decline affect housing choices of older adults. Granbom et al. (2019) found that residential mobility among community living older adults was related to the challenges in accessibility in their home environment, whereas moving into residential care was more a result of health-related factors and living alone. An apartment building may become an attractive housing option when people want to prepare for their old age (Andersson et al., 2019). One-floor dwellings, having a kitchen, bedroom, and bathroom on the same floor, support older adults who have challenges in mobility. Then again, existing apartment building stock may have many shortages. Many old apartment buildings lack lifts. The study by Brim et al. (2021) revealed that older adults self-report hindrances related to the immediate surroundings (front door, stairwell, etc.) and their home environment (flooring, bathroom, and thresholds).

### Safety Regulations and Solutions

The safety regulations and assessment of risks in the home environment for older adults are heavily based on aspects of physical safety (Kivimäki et al., 2019). Safety in housing construction is realized through structural and fire safety, space functionality as well as physical and sensory accessibility. Older adults are considered the largest risk group in building fires due to their functional limitations, reduced ability to act in building fire, and evacuate (Gjøsund et al., 2016). In Finland, building regulations and fire classifications for care facilities are applied in housing construction targeted at older adults and other vulnerable groups. Due to fire safety, some design features are standardized, and choice of surface materials is limited, which may make the environment look institutional or plain. The possibility to furnish or provide resting places in common-use spaces like building entrances or corridors is limited because of fire regulations. Moreover, safety solutions like heavy fire doors can create hindrances to older adults’ independent mobility. This may affect the usability and comfort of the living environment.

Safety and usability at home relate to the dimensioning of spaces, accessibility, and to the choice of materials, furnishing, and lighting. In hazard events, older people themselves have reported that narrow passageways, stairs, and motorized scooters making evacuation difficult (Coty et al., 2015). The Finnish National Rescue Association provides a checklist for home safety for vulnerable resident groups. In addition to the building and technical aspects of safety, it also includes factors related to the functioning capacities of the resident. The recommended technical fire safety solutions for vulnerable groups and older adults include stove guards, smoke alarms, and fire sprinklers. Furthermore, personal alarms and digital technology are strategies for enhancing safety. Coty et al. (2015) point out that some older adults have limited financial resources to improve home fire safety.

### Feeling Safe in the Community

Physical safety and accessibility are unquestionably important. In addition to physical safety, older adults living in assistive housing have been reporting feeling safe because of the presence of staff members and other familiar people around them (Lindahl, 2015; Slettebø, 2008). For community living seniors, local services and social cohesion may act as a buffer against the negative effects of living alone (Cramm et al., 2013). Runefors et al. (2021) study indicated that for older adults living in urban areas, proximity of neighbors and short response time of first responders were significant predictors of survival in building fires. They found that almost 20% of evacuations of older adults were by neighbors; however, the share was lower for persons aged 80 years and older. Similarly, Nilson et al. (2020) found that living with others, whether younger or of the same age, is correlated with lower fire mortality rates. As the number of older adults living alone at home increases, the efforts to improve the community cohesion are important.

## Methods and Study Sample

### Data Collection

Utilizing a case study approach this paper presents reflexions on safety in the context of a building fire in a senior housing in Finland. The qualitative research methods and hermeneutic approach used in this study included semi-structured interviews with five independently living older adults who had recently lived through the building fire. The data from individual interviews, a follow-up group discussion with the informants, and observations on site enabled to increase knowledge of the housing safety for older adults. The study focus was on the housing and the built environment.

The case study is a newly built senior housing complex (built in 2018) that took fire in the summer of 2020. Luckily, all 170 residents of the senior housing complex were evacuated safely, but the damages to the buildings were important. The senior housing was composed of three buildings: a central building with residential care for older adults and two adjacent buildings with rental and right-of-occupancy housing for seniors. Right-of-occupancy is presenting an alternative between owner-occupied and rental housing. After the fire, the independently living older adults were provided with temporary dwellings for the renovation period, approximately 10 months. They were relocated within the city. In Spring 2021, during the first contact for interviews, the residents had been living back home for approximately 3 weeks.

The study used purposeful sampling. Potential informants among residents of one of the rental apartments were recruited for the study in collaboration of their landlord, a non-profit organization. Invitations to take part in the study were put on the noticeboard of the rental buildings. As the topic was sensitive, the residents were asked to contact themselves the researcher in case they were willing to contribute. Five persons out of fifty-two residents volunteered for the interview. They were living in the same apartment building, but the relationship between informants did not come up during the individual interviews. In the follow-up meeting, it turned out that the two persons participating were acquaintances. Informants were independent living seniors and older adults, from 61 years to 94 years old, capable of providing informed consent. Two of the informants were living with a partner, and the three others were living in single-person households (Table 1). Four of them were retired from work life, and one of them had a part time occupation. All interviewees were living in two-room apartments targeted to seniors (age limit +55) with a large balcony.

**Table 1.** Interviewees Profiles (Participants’ Pseudonyms).

Martha	Helen	Rosemary	Iris	Peter
Age 75	Age 82	Age 94	Age 61	Age 76
Living alone	Living alone with her cat	Living alone	Living with her partner	Living with his partner

Due to the COVID-19 restrictions in the spring of 2021, face-to-face meetings and interviews, as well as site visits, were not possible. After giving their written informed consent, the time for interview was decided together with the participants. As the interviewees had no access to remote conferencing systems, the author interviewed four of the informants by phone. One of the interviewees (Iris) preferred to answer in written, as she felt the topic too overwhelming. The individual interviews took 45–70 minutes and were audio recorded. The author carried out the Finnish transcription of the interview and the condensed transcription in English.

The study used semi-structured interviews. The semi-structured interviews were based on a literature review on meaning of home and well-being. The questions were formulated and discussed together with a group of researchers. They were adapted from one of the previous studies carried out by one of the researchers (Adalgeirsdottir, 2021, p. 49). The aim was to have simple and short questions that were easy to answer. The main goal was to listen attentively to the informants with empathy with understanding of the sensitivity of the topic. The open-ended questions were related to home and community, and experiences during the emergency and recovery phases (Table 2). The semi-structured interview was aiming to steer the conversation on the role of the built environment in hazards and to collect similar types of information from each participant (Kallio et al., 2016). A follow-up group discussion was organized in December 2021 on-site, but only two of the interviewees were able to attend. On the occasion, the researcher had the possibility to visit the site with one of the informants and see one of the residents’ photos of the building fire.

**Table 2.** Themes of the Semi-structured Interviews.

Theme	Type of questions
Living environment	Can you describe your home?
	Can you describe your community?
The emergency situation	How did the fire affect your home?
	What did you take with you?
	Where did you stay after fire?
The recovery phase	What has changed in your living environment?
	What could have been done differently?

### Data Analysis

This descriptive study used thematic content analyses. It included the ways older people were talking about the hazard and about the changes in their living environment. The overall objective of the hermeneutic approach (Steeves, 2000, p. 47) was to understand the factors related to resilience and safety. The larger framework of this study was the phenomenon of population aging and future urban resilience. The sample was small, and the five interviews were analyzed manually using thematic content analysis. In the first phase, experiences of the fire and reflexions on home and community reported by each informant were analyzed. In the second phase, the narratives of the hazard were analyzed and compared in the time frame: before, during, and after fire. In the end, the author interpreted the data in the bigger framework of aging and housing safety. The themes related to physical and social safety were reoccurring in each interview and were chosen in this paper to report the findings of the study. The informants lived in an apartment building that had only minor damages. This together with the low number of participants may influence the applicability of results to other cases.

## Results

To support the daily living and social activities of seniors and older adults, new communal housing solutions have been built. The informants of this study were living in one of the newly built senior housings. The reason for moving to the senior housing reported by the informants was access to an age-friendly safe living environment with social support. They reported their experiences of the loss of community and control over their own home due to the building fire. The damages to their homes could be repaired, but the community rebuilding will require time and common-use spaces.

### Housing and Evacuation Phase

In the first phase, the interviewees were asked to describe their home and length of residence. All interviewees reported to had been moving the newly built senior housing two years prior to the fire. They lived in subsidized rental apartments that were targeted to people in the age cohort 55+, which one of interviewees described as one’s “end-of-life home.” The interviewees were describing the cosiness of their own apartment. At the same, the common-use spaces emerged in each description as extension of one’s own home. The common-use living room, restaurant, spaces for hobbies, and outdoor spaces came up in all interviews.

“We wanted to move to a house where all services are close. I also fell in love with the idea of possibility of urban farming. My commuting was shortened, and my husband was getting retired” (Iris).

The building was constructed according to the current accessibility standards and was designed to support older adults’ functional needs. Moreover, reports for fire safety showed that the apartments and balconies were equipped with fire sprinklers. Nevertheless, the starting point of the fire was a candle placed on the outer part of a balcony. The fire was able to grow before the sprinklers went on and it spread to all three buildings through roof structures due to defective fire-separating elements. The central building with residential care was destroyed by the fire. The independently living residents of the two adjacent apartment buildings were evacuated because of the damages due to fire and smoke.

Secondly, the informants were asked to describe the course of the hazardous event. It occurred that the building fire was in the evening news before all residents were alerted and evacuated. Helen first heard about the fire in the radio, and Rosemary had a call from a relative informing her about it in the news in television. Martha and Iris reported that the view from their window to the scene of people gathering on the street alerted them to leave the building. All interviewees reported having been able to exit the building by themselves. They reported that several neighbors had mobility challenges, were using rollators, and had difficulty in taking the stairs. They were carried downstairs by the first-rescue team.

**Figure 1.**
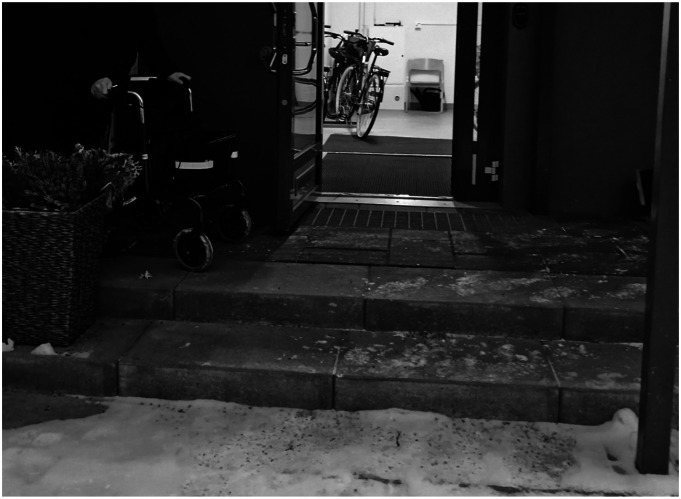
Doors hinged on the wrong side are hindering exit in emergency (photo, Verma, I).

Peter reported that a few accidents and falls had occurred at the building entrance when older residents rushed out of the building. In a great hurry, at the entrance, older adults with rollators took the stairs instead of an accessible ramp, as the stairs was visually the most direct way out of the building. During the site visit, the author observed the entrance door opening on the access ramp blocking the evacuation route (Figure 1).

Building design, building materials, and a fire exit safety plan increase safety. The distance to fire exits and the requirements for staircase are regulated. This case study confirmed that lifts are important in daily coping for older adults, but they are not relevant in the evacuation process. Therefore, evacuation of a large group of vulnerable people is difficult when the lifts cannot be used in fire. A safe, easy-to-use staircase with handrails on both sides can make evacuation safer. Two-way dimensioned traffic in staircases and large intermediate landings enable rescue personnel and other people to pass. Moreover, stairs with clear contrasts and glare-free even lighting may enhance the visual perception. Additional technical solutions like emergency lighting system and photoluminescent guidance provide a safety-enhancing element during a fire or power outage. At entrance, a large even landing with direct access to an accessible ramp makes the emergency evacuation safer. The door opening direction is important in daily use as well as in evacuation.

### Living in Temporary Housing

The renovation period for the apartment buildings was eight to ten months. The informants were asked how they managed and where did they live after the fire. The informants described that during this period residents were dispersed in various parts of the city in temporary dwellings (Table 3). They all had been living in several places. Iris reported having lived in seven different locations during her nine-month evacuation period.

**Table 3.** Resident Participants Temporary Housing Paths.

Martha	Helen	Rosemary	Iris	Peter
- Two nights with one of her children	- Two nights with her children	-Two nights at one relative	-Four nights in a hotel and with friends	-1.5 weeks in a hotel
-Two weeks with another child	-Two weeks in a hotel (without her cat)	-One month with another relative	-Short stays in various rental apartments	-1.5 months in an apartment hotel
- In a subsidized rental apartment	- An apartment hotel (which accepted cats)	-In a rental apartment	-8 months in a rental apartment	-In a rental apartment
	- In a rental apartment			

The informants were asked what makes a dwelling a home and did they take something with them to the temporary dwelling. The results show the meaning of personal objects for people.

“Of course, what makes a home? Everything I have, everything a poor grandma has, all that a poor grandma has…”(Rosemary).

They told that approximately two weeks after the fire, residents got the permission to collect some of their personal belongings from home. At the time, they did not have information about the length of the renovation period. Informants reported having taken, in addition to clothing, a tablecloth, cutlery, and coffee cups with them to make the temporary dwelling homey and esthetic. Moreover, Helen reported having taken the radio, which was on day and night during the stay in temporary dwelling. It can be noticed that as she first heard about the fire through radio, the sound of the radio may have increased her feeling of safety.

### Role of Public Facilities in the Evacuation Process

**Figure 2.**
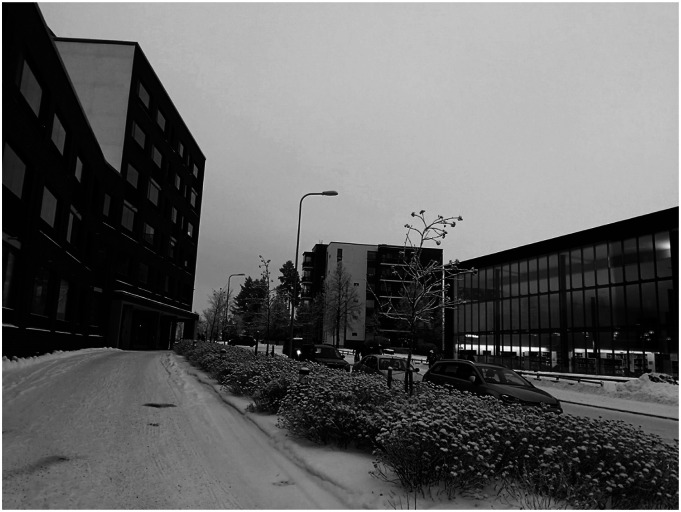
Easy access to a library building (on the right) helped in the evacuation of frail older adults living in the senior complex (on the left) (photo, Verma, I).

The location of the building in the urban structure influenced the evacuation process. The informants described how in the first phase, an open space in front of the building was used for evacuation of residents. In the second phase, a library and a school building on the opposite side of the road functioned as the first shelter for rescue (Figure 2). The proximity of the public facilities had a key role as the center for evacuation, assistance, and information. First rescue personnel, the Red Cross, and other volunteers were able to use these public facilities for evacuation.

The evacuation of residents from the central building with residential care was challenging because some of them were bed-bound and needed to be carried out. Residents from the senior housing interviewed in the local media reported that the doors of the library building were not wide enough for hospital beds; therefore, the bed-bound residents from the residential care were evacuated to the adjacent school building (Kivinen, 2020). The results of this case study show that locating housing for seniors in the proximity of public facilities may facilitate rescue operations. Moreover, the considerations for the accessibility and dimensioning, for example, the entrance doors of public facilities, are relevant in daily use as well as in emergencies.

### Role of Common-Use Spaces for the Community

Interviewees reported having enjoyed the social activities, passively or actively, in the senior community. The informants were asked to describe their community. They told how the built environment and easily accessible communal spaces contributed to the social environment and promoted community cohesion. Rosemary reported that she had not been actively looking for a conversation, but other people were seeking her company. She had made new relationships. The restaurant and common-use premises had been the main places for daily encountering.

Martha and Helen reported to have celebrated birthdays and other festivities together with neighbors and play cards and board games with them in the common-use spaces. These activities supported residents’ mobility and daily activity. They described that many neighbors were willing to give peer support and share leisure activities. Helen reported to have got a weekly ride to the swimming pool with a neighbor. The access to outdoor spaces was enabling walking, barbecuing, and working in the garden with neighbors. Due to fire, all shared-use premises having a central role in community building were destroyed. During the time of the interviews, these premises were still under renovation and not in use. Residents felt this to be slowing down the rebuilding of the community. They found that the peer support and connection with neighbors were resources for their individual recovery from the crises.

“We had the dining room, common living room, an awfully nice community. We had everything, ‘tikkupeli’ and everything. ‘Tikkupeli’ is a kind of memory game. It is so gripping, and you can exercise your memory…The fire and COVID-19 knocked us off our feet. They have promised ready for Christmas… well, we will see. And not everybody is returning to the house” (Helen).

Older adults interviewed for this study were emphasizing the importance of the community for the safety and feeling of safety. In the first phase, the senior community with shared facilities and secure living environment had been an important pull factor to move to the senior housing. During the period of residence, the presence of other people and connection to the community were enhancing their feeling of safety. The community had been a source of support in daily life as well as during the fire. The interviewees described how they had been themselves participating in alerting and assisting their neighbors to evacuate.

The biggest loss reported by each interviewee was the loss of the community. During the renovation period, the residents reported having had only a few occasions to meet their old neighbors from the senior housing. Peter who had been active in residents committee regretted having lost touch with old neighbors and not being able to share experiences with them. Others complained a lack of common meeting space during the renovation phase. According to the results, access to common-use spaces may have enabled to maintain social relations and peer support during the renovation process.

### Personal Coping and Recovery Phase

People have different personal capacities to adapt to change and overcome hazards in their lives. The interviewees reported having gone through many personal adversities and losses during their life course. Their personal attitude and resilience, based on past experiences, helped them to recover from the crises. Martha was comparing her personal hardships, that were many, to those of the previous generations.

“I have been thinking that way... I have not got round to write it down: My grandpa and grandma were evacuated twice… For those (residents) over 90 and have experienced the war, it must have been a tough place. You just have to survive” (Martha).

Interviewees reported that the support of relatives, friends, neighbors, and pets helped them to survive emotionally. Two of the interviewees described a positive impact of building up trust between a group of friends after the fire. Helen said that she would have not been able to overcome the situation without her cat. Rosemary seemed reluctant to ask for any formal help from “*strangers*” and was proud of managing the crises by herself. On the other hand, she told that she did not know where to ask for help. Peter felt that the older residents would have needed more practical assistance and economic advice to meet their basic needs. Some of them reported that all their personal objects including phone, bank card, and medication remained in the apartment when they were evacuated. Helen told that during her stay in the temporary dwelling the practicalities, how to buy food and medication, where to pick up the mail, and how to do the laundry were the major concerns. Moreover, for low-income older adults, living in subsidized apartments, the uncertainty of the rent allowances and compensations by insurance caused additional emotional and financial stress.

The interviewees were heavily criticizing the lack of information about the renovation process and timeline. As the residents were renters, they were not involved as partners in the renovation process. Iris reported to had had to rely on, often erroneous, information received through the media. They experienced feeling left out in matters that were concerning their own homes. The renovation included, for example, construction of an additional fire protection on the balconies which had visual impact. At the time of moving back, seeing the modifications carried out in their homes left them disappointed. Peter considered moving to the unfinished construction site a “*rough*” and traumatic emotional experience. He described the view from his apartment to the construction site as being “*desolate.*” Moving back raised mixed feelings among all the interviewees.

“Adjustment has been very difficult for some people. ‘What if something like this happens again?’ […] It is like a dispute with neighbours; even if you settle the dispute, the confidence has gone” (Peter).

The possibility to move back home was an emotional and overwhelming event. The new apartment building had been in their images the secure “end-of-life home.” Therefore, the fire was a big shock, which was not foreseeable. The fire challenged their trust and feeling of security. Approximately one-third of old neighbors had moved out and new neighbors moving in. Residents felt that the building up of the community had to start all over again. Residents were saddened about the loss of their social networks but were looking for the facilities to open again to build up new relations. Due to renovation, the common-use premises were not in use and made the community rebuilding challenging.

## Discussion

The results of this study confirm earlier findings that the motivations to move to a senior housing are related to feeling of security and having support from the community (Lindahl, 2015; Rusinovic et al., 2019). The community building is related to easy access to common-use spaces and public facilities. This study shows that they have an important role in daily activities, in emergency and in recovering from adversity. This study provides useful knowledge and user experience related to hazardous events in aging cities. The result indicates that maintaining social networks and having spaces to meet may support the community rebuilding process after hazards.

The older adults form a heterogeneous group of people with various competencies, functional capacities, and housing conditions. As majority of older adults live in ordinary housing, the safety and user friendliness become important factors in housing construction. The vulnerability of an individual varies in time, depending on one’ social and physical environment as well as individual capabilities. Gjøsund et al. (2016) argue that measures to improve the safety of vulnerable people are not only related to fire prevention barriers but also measures that are decreasing the vulnerability of older adults and improving their quality of life in general. Brim et al. (2021) point out the need for financial support for maintenance and repairs of older adults’ current homes. The housing environment can become a platform for safe, physically, and socially active aging.

Earlier studies indicate that older adults themselves relate safety to the ability to handle everyday activities to their own satisfaction, having someone to rely on for help, and feeling at home in their dwelling as well as in the surroundings (Lindahl, 2015; Petersson et al., 2012). Our study confirmed that older adults were satisfied with their age-friendly apartments and wanted to maintain the control over their home environment. Interviewees reported that their living environment with accessible common-use premises increased the social activity, neighborliness, and sense of security. Kloseck et al. (2014) argue, however, that moving to a retirement community may generate false expectations of reduced exposure to hazards and a sense of security. Our study indicated that the expectation of a safe “end-of-life dwelling” may have aggravated the interviewees’ experiences of loss.

To build up resilience and help people to recover from future hazards related to the built environment, the safety needs to include the social and emotional aspects of safety as well. The quality, safety, and accessibility of the environment are linked to the possibility to stay active. The built environment can provide places for community building and contribute to social cohesion and feeling of safety. In turn, a high level of safety may increase older adults’ satisfaction with their social networks (Kemperman et al., 2019). The proximity of public facilities can enhance the possibilities for social activities and lead to inclusion of older adults in their community. Inclusion of people of different ages and capacities in housing areas may increase the resilience and improve the safety in hazards.

To conclude, the case study shows that safety protocols and rescue processes were professionally and well organized, and no lives were lost. However, the older adults reported to have needed more practical, social, and emotional support after the fire and during the renovation period. As Peter said, older adults are hesitant to ask for support, but at the same, they need the support. The temporary accommodation not only needs to provide a suitable physical environment but also social and emotional support to older adults. The ability to maintain established social contacts may protect against depression and other negative psychological impacts after crises (Evans, 2010). Community cohesion and peer support are important in the process of recovery from adversity. It should be encouraged through the built environment. In the case of building fire, older adults would have benefited from access to a communal space to meet their peers and share their experiences. After crises, the repairing and rebuilding of social infrastructure to maintain social contacts should be prioritized. Moreover, it may have helped to regain trust and security.

The modifications to the older adults’ homes were carried out without their participation. They felt have lost control over their home environment. Furthermore, they reported to have been lacking information about the renovation process. People have tendency to do things for older adults, without involving them in the planning or decision processes. This decreases their feeling of safety and self-worth. Gustavsson et al. (2021) point out, that for fire safety, even though the service personnel may inform and suggest changes and technical solutions related to safety, the resident decides, which changes to make. In addition, the older person needs to be active in applying for implementation of such solutions.

Previous findings show that many older people have more experience and skills to overcome adversities in their life than younger generations (Kekki et al., 2021). On the other hand, the experience of a disaster may increase insecurity and fear that a similar event will occur in the future (Sobkow et al., 2020, p. 123). Older adults may be skeptical about the availability of help and unsure if the services would satisfy their needs and who will define what are their actual needs (Gunnarsson, 2009). A broader view on safety related to housing and living environments may support older adults’ daily living and their resilience.

## Supplemental Material

Supplemental Material - Safety in Housing for Older Adults—A Qualitative Case Study Supplemental Material for Safety in Housing for Older Adults—A Qualitative Case Study by Ira Verma in Journal of Applied Gerontology.
