# Supplementary material for: Safety in Housing for Older Adults—A Qualitative Case Study
Source: J Appl Gerontol. 2024 Jul 18;43(12):1914–23. doi: 10.1177/07334648241260212 (PMC11552200; doi:10.1177/07334648241260212)
Supplement: Supplemental Material - Safety in Housing for Older Adults—A Qualitative Case Study [file sj-pdf-1-jag-10.1177_07334648241260212.pdf]

COREQ (COnsolidated criteria for REporting Qualitative research) Checklist

A checklist of items that should be included in reports of qualitative research

Topic	Item No.	Guide Questions/ Description	Author Responses
**Domain 1: Research team and reflexivity**			
Personal Characteristics			
Interviewer/facilitator	1	Which author/s conducted the interview or focus group?	Ira Verma
Credentials	2	What were the researcher's credentials? E.g. PhD, MD	D.Sc. in Architecture
Occupation	3	What was their occupation at the time of the study?	Senior scientist
Gender	4	Was the researcher male or female?	Female
Experience and training	5	What experience or training did the researcher have?	15 years experience on qualitative research with older adults
Relationship with participants			
Relationship established	6	Was a relationship established prior to study commencement?	No relationships prior to study
Participant knowledge of the interviewer	7	What did the participants know about the researcher? e.g. personal goals, reasons for doing the research	The participants got a written description and objectives of the study. The researcher explained the overall aim of the study: how to better prepare for future crises.
Interviewer characteristics	8	What characteristics were reported about the inter-viewer/facilitator? e.g. Bias, assumptions, reasons and interests in the research topic	The interviewer explained that she was an architect and researcher, interested in the built environment. The interview was explained to be part of a larger study on future resilience of cities. The researcher also told, that all information will be kept confidential and participants have the possibility any time to withdraw from the study.
**Domain 2: Study design**			
Theoretical framework			
Methodological orientation and Theory	9	What methodological orientation was stated to underpin the study? e.g. grounded theory,	The paper presents a qualitative case study. Main method used was a semi-structured interview. The study uses thematic content

		discourse analysis, ethnography, phenomenology, content analysis	analyses.
Sampling	10	How were participants selected? e.g. purposive, convenience, consecutive, snowball	The sampling was purposive. The residents who had been living in the rental building that was damaged in the fire prior and after the hazardous event were potential informants.
Method of approach	11	How were participants approached? e.g. face-to-face, telephone, mail, email	The participants were first approached by an open invitation letter at the building entrance lobby. The informants were asked to contact the researcher themselves to volunteer. The interviews were carried out by phone. Later a face-to-face follow up meeting was organized.
Sample size	12	How many participants were in the study?	Five older adults
Non-participation	13	How many people refused to participate or dropped out? Reasons?	No dropped outs, but only 5 out of potential 52 persons volunteered.
<u>Setting</u>			
Setting of data collection	14	Where was the data collected? e.g. home, clinic, workplace	The data was collected on the university computer and stored and processed following the university data protection policy.
Presence of non-participants	15	Was anyone else present besides the participants and researchers?	No
Description of sample	16	What are the important characteristics of the sample? e.g. demographic data, date	Participants were older adults from 61 to 94 years old. Interviews took place in May 2021.
<u>Data collection</u>			
Interview guide	17	Were questions, prompts, guides provided by the authors? Was it pilot tested?	An interview guide was prepared based on a previous study.
Repeat interviews	18	Were repeat interviews carried out? If yes, how many?	A follow-up group interview was organized 6 months after interview.
Audio/visual recording	19	Did the research use audio or visual recording to collect the data?	The interviews were audio recorded.
Field notes	20	Were field notes made during and/or after the interview or focus group?	Photographs and field notes were made during the visit on site.

Duration	21	What was the duration of the interviews or focus group?	Each interview took 45 to 70 min.
Data saturation	22	Was data saturation discussed?	
Transcripts returned	23	Were transcripts returned to participants for comment and/or corrections?	The interview results were discussed in the follow-up meeting
**Domain 3: analysis and findings**			
Data analysis			
Number of data coders	24	How many data coders coded the data?	On this material one, two other similar cases were carried out with two other researchers. The joint results will be presented in an other paper.
Description of the coding tree	25	Did authors provide a description of the coding tree?	A mental map was prepared
Derivation of themes	26	Were themes identified in advance or derived from the data?	The themes of home and community were identified from literature, the themes of physical and social safety derived from the data.
Software	27	What software, if applicable, was used to manage the data?	Not applicable
Participant checking	28	Did participants provide feedback on the findings?	Yes, on the follow-up meeting
Reporting			
Quotations presented	29	Were participant quotations presented to illustrate the themes/findings? Was each quotation identified? e.g. participant number	Quotations have not been presented to the informants. They are identified by each informant.
Data and findings consistent	30	Was there consistency between the data presented and the findings?	The data confirmed findings from literature and from observations.
Clarity of major themes	31	Were major themes clearly presented in the findings?	Major themes were related to the ageing population and urban resilience
Clarity of minor themes	32	Is there a description of diverse cases or discussion of minor themes?	The study is about one case experienced by five individuals. Minor themes are related to personal differences.

Developed from: Tong A, Sainsbury P, Craig J. Consolidated criteria for reporting qualitative research (COREQ): a 32-item checklist for interviews and focus groups. *International Journal for Quality in Health Care*. 2007. Volume 19, Number 6: pp. 349 – 357

Additional Guidelines for Completing the COREQ Checklist for *Journal of Applied Gerontology*:

- This checklist will be published online as supplementary material and we require it to be in the form of a publishable table. Please make sure that material does not bleed outside of cells, etc.
- This checklist is designed to direct readers to relevant material in the manuscript. Where applicable, please direct readers to various sections of the manuscript, such as a Methods section, Conceptual Framework, table or figure. Pages may shift during the publication process so please avoid directing readers to specific page numbers.
- This checklist also is designed to supplement information that may not be reported in the text and/or provide additional details related to information that is reported in the text.

Once you have completed this checklist, please save a copy and upload an anonymized version of it as part of your *Journal of Applied Gerontology* submission. DO NOT include this checklist as part of the main manuscript document. It must be uploaded as a separate supplemental file. If the paper is accepted, a non-anonymized version should be provided with the final submission of the main manuscript.
